# Identification of an Individualized Metabolism Prognostic Signature and Related Therapy Regimens in Early Stage Lung Adenocarcinoma

**DOI:** 10.3389/fonc.2021.650853

**Published:** 2021-04-28

**Authors:** Junjie Hu, Huansha Yu, Liangdong Sun, Yilv Yan, Lele Zhang, Gening Jiang, Peng Zhang

**Affiliations:** ^1^ Department of Thoracic Surgery, Shanghai Pulmonary Hospital, Tongji University School of Medicine, Shanghai, China; ^2^ Experimental Animal Center, Shanghai Pulmonary Hospital, Tongji University School of Medicine, Shanghai, China; ^3^ Central Laboratory of Thoracic Surgery, Shanghai Pulmonary Hospital, Tongji University School of Medicine, Shanghai, China

**Keywords:** lung adenocarcinoma, early stage, metabolism genes, prognostic signature, adjuvant therapy

## Abstract

**Objective:**

The choice of adjuvant therapy for early stage lung adenocarcinoma (LUAD) remains controversial. Identifying the metabolism characteristics leading to worse prognosis may have clinical utility in offering adjuvant therapy.

**Methods:**

The gene expression profiles of LUAD were collected from 22 public datasets. The patients were divided into a meta-training cohort (n = 790), meta-testing cohort (n = 716), and three independent validation cohorts (n = 345, 358, and 321). A metabolism-related gene pair index (MRGPI) was trained and validated in the cohorts. Subgroup analyses regarding tumor stage and adjuvant chemotherapy (ACT) were performed. To explore potential therapeutic targets, we performed *in silico* analysis of the MRGPI.

**Results:**

Through machine learning, MRGPI consisting of 12 metabolism-related gene pairs was constructed. MRGPI robustly stratified patients into high- *vs* low-risk groups in terms of overall survival across and within subpopulations with stage I or II disease in all cohorts. Multivariable analysis confirmed that MRGPI was an independent prognostic factor. ACT could not improve prognosis in high-risk patients with stage I disease, but could improve prognosis in the high-risk patients with stage II disease. In silico analysis indicated that B3GNT3 (overexpressed in high-risk patients) and HSD17B6 (down-expressed in high-risk patients) may make synergic reaction in immune evasion by the PD-1/PD-L1 pathway. When integrated with clinical characteristics, the composite clinical and metabolism signature showed improved prognostic accuracy.

**Conclusions:**

MRGPI could effectively predict prognosis of the patients with early stage LUAD. The patients at high risk may get survival benefit from PD-1/PD-L1 blockade (stage I) or combined with chemotherapy (stage II).

## Introduction

Lung cancer is the leading cause of cancer-related death worldwide (1), and early stage lung cancer accounts for about 17% (2). Lung adenocarcinoma (LUAD) is the most common histologic subtype of lung cancer (3). Surgical resection plus lymph node dissection or sampling is the standard treatment for stage I LUAD (4). However, some patients will still suffer from disease relapse and death, and the 5-year overall survival ranges from 68 to 92% (5). According to the National Comprehensive Cancer Network (NCCN) guidelines, adjuvant systemic treatment is only considered for high-risk patients (4). The benefit of adjuvant systemic treatment for stage I LUAD remains controversial.

Biomarkers, especially gene expression, in tumor tissues are reliably related to cancer prognosis and survival (6–8). Thus, identifying the molecular features that may lead to worse prognosis may have clinical utility in offering adjuvant therapy to a subgroup of patients at high risk. The availability of large-scale public cohorts with gene expression data provides an ideal resource to identify a more individualized prognostic signature for LUAD.

Reprogramming of energy metabolism is an emerging hallmark of cancer (9) and recently has been proved to be involved in lung cancer initiation, progression, and drug resistance (10–13). Metabolic phenotypes can also be exploited to image tumors, provide prognostic information, and treat cancer (14). Therefore, understanding the metabolism characteristics by gene expression-based algorithms may be helpful for screening the patients at high risk. However, the molecular characteristics of tumor metabolism remain to be comprehensively explored regarding their prognostic potential in early stage LUAD.

In this study, we integrated multiple cohorts with gene expression profiles to develop and validate an individualized prognostic signature for early stage LUAD from metabolism-related gene pairs (MRGPs). We then explored the potential therapy regimen for the patients at high risk, which may be utilized in clinical. Further, to leverage the complementary value of molecular and clinical features, we integrated the metabolism signature with clinical factors to improve the predicted accuracy for overall survival (OS).

## Methods

### Patients and Datasets

This study was approved by the Ethic Committee of Shanghai Pulmonary Hospital. We retrospectively analyzed the gene expression matrixes and corresponding clinical characteristics from 22 public datasets (**Supplementary Table S1**), including 17 microarray and two RNAseq datasets from the Gene Expression Omnibus (GEO) database (https://www.ncbi.nlm.nih.gov/geo/), one RNAseq dataset from the Cancer Genome Atlas (TCGA) database (https://portal.gdc.cancer.gov), one microarray dataset from the ArrayExpress database (https://www.ebi.ac.uk/arrayexpress/), and one RNAseq dataset from the OncoSG database (15) (https://src.gisapps.org/OncoSG/). The patients were included according to the following criteria: (1) lung adenocarcinoma, (2) stages I–II, (3) available OS information. The patients were excluded if they met any of the exclusion criteria: (1) non-adenocarcinoma or the pathologic subtypes were unknown, (2) stage III or IV or unknown, (3) lack of OS information, (4) received neoadjuvant therapy. The gene expression matrix of normal lung tissue was downloaded from the Genotype-Tissue Expression (GTEx) database (https://www.gtexportal.org/home/). The entire tumor datasets were divided into meta-training, meta-testing, and three independent validation cohorts (TCGA, GSE68465, and GSE72094) (**Supplementary Figure S1**).

### Data Process

All the expression level of microarray datasets was transformed by log2. For all the datasets of RNAseq, the fragments per kilobase million (FPKM) level was used as the expression value and log2(FPKM+1) transformed. If there were duplicate genes in each dataset, the mean value was calculated by the *avereps* function from the *limma* R package.

### Construction of the MRGPI

**Figure 1:**
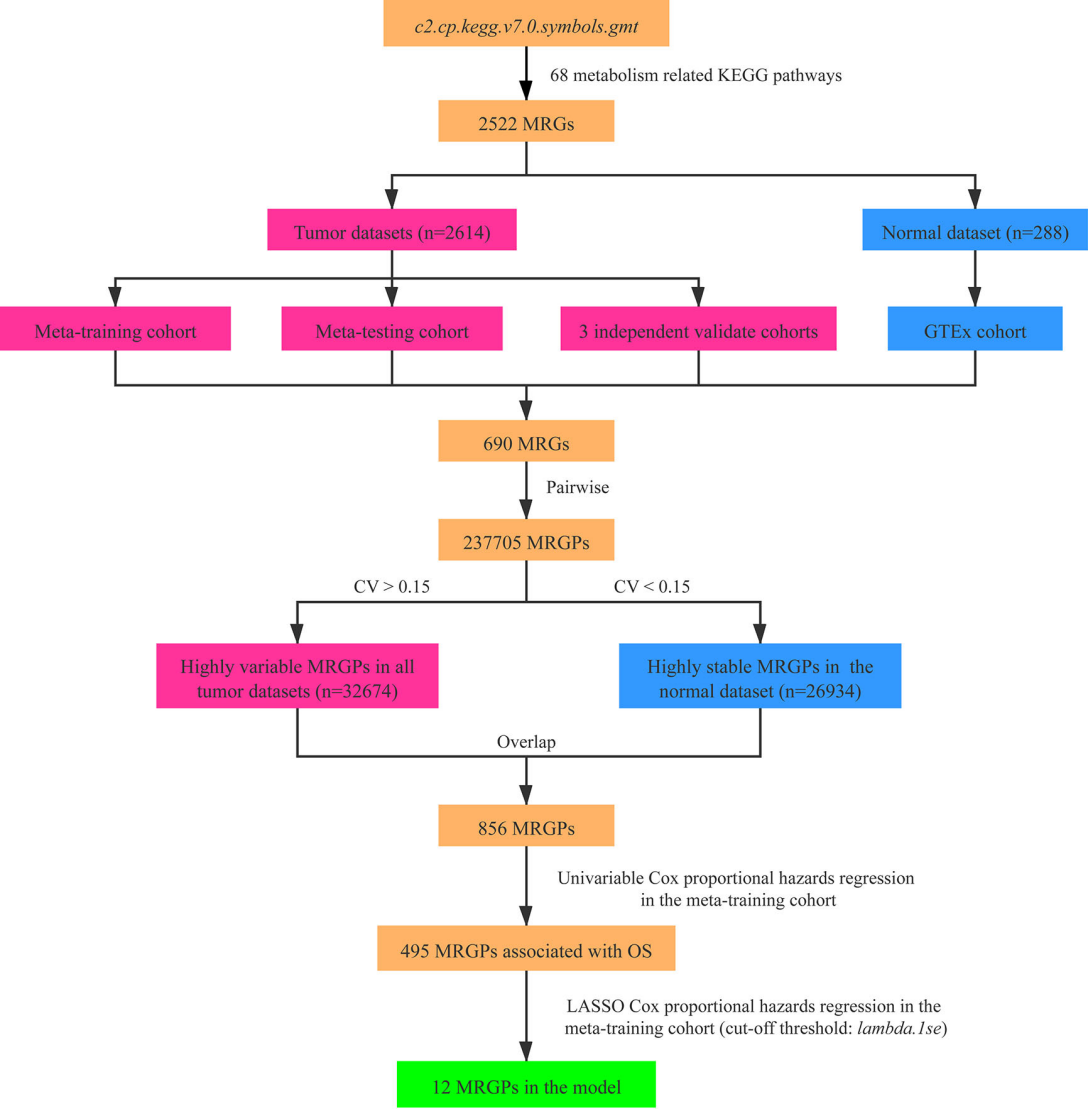
Flowchart of the construction process of MRGPI. CV, coefficient of variation; GTEx, the Genotype-Tissue Expression; LASSO, least absolute shrinkage and selection operator; MRGs, metabolism-related genes; MRGPs, metabolism-related gene pairs; OS, overall survival.

As shown in the **Figure 1**, we constructed a prognostic signature by focusing on metabolism-related genes (MRGs). From the *c2.cp.kegg.v7.0.symbols.gmt* dataset that was downloaded from the Gene Set Enrichment Analysis (GSEA) website (https://www.gsea-msigdb.org/gsea/index.jsp), 2,522 MRGs from 68 metabolism related Kyoto Encyclopedia of Genes and Genomes (KEGG) pathways were identified. Of the 2,522 MRGs, 690 MRGs were available in all datasets. The gene expression value underwent pairwise subtraction to generate a score for each metabolism-related gene pair (MRGP): MRGP score = expression value of MGP 1 − expression value of MGP 2. The score represented the log2 fold change of MGP 1 relative to MGP 2.

To screen the representative MRGPs in tumor, we identified the MRGPs that were highly variable [coefficient of variation (CV) > 0.15] in all tumor datasets and highly stable (CV < 0.15) in the normal cohort. Then the univariable Cox proportional hazards regression was used to select prognostic MRGPs in the screened MRGPs (*survival* R package). Finally, to minimize the risk of overfitting, a cox proportional hazards regression model combined with the least absolute shrinkage and selection operator (LASSO) was applied to identify the most important prognostic MRGPs (*glmnet* R package). The optimal values of the penalty parameter *λ* were determined by 10-fold cross-validations at 1 SE beyond the minimum partial likelihood deviance in the meta-training cohort. Based on the selected MRGPs from LASSO Cox regression model, the metabolism-related gene pair index (MRGPI) for each patient was constructed: MRGPI = $\text{MRGPI}=\Sigma_{i}^{n}\;MRGP_{i}\;score\times Coefficient_{i}$. To separate patients into low- or high-risk groups, the optimal MRGPI cutoff value was determined using the *surv_cutpoint* function of the *survminer* R package.

### Validation of the MRGPI

The predictive value of MRGPI for OS was evaluated in the meta-training, meta-testing and three independent validation cohorts. As described in a previous study (6), the pathologic stage was treated as continuous variable by the following converting approach: IA was coded as 1, then IB as 2, I as 1.5, I–II as 2.5, IIA as 3, IIB as 4 and II as 3.5. The univariable Cox regression model was used to evaluate the prognostic value of age, gender, smoking history, stage and MRGPI (as continuous and binary form, respectively). The multivariable Cox regression model was used to evaluate the independent prognostic value of MRGPI. Subgroup analysis was performed according to the stage.

### DEGs and Gene Ontology Analysis

The gene expression differences between high and low risk were compared using the *limma* package, and genes with |log fold change| > 1 and false discovery rate adjusted P value <0.05 were considered to be significant differentially expressed genes (DEGs). To gain biological understanding of the MRGPI, we conducted an enrichment analysis of its component MRGs using the *clusterProfiler* R package. FDR-adjusted P <0.05 was used to select statistically significant gene sets.

### Profiling of Infiltrating CD8 T Cells

To analyze the tumor immune microenvironment, a dataset of single cell RNAseq (scRNA-seq) with annotated cell types (16) (GSE131907) was downloaded from the GEO database. There were nine samples of stage I–II LUAD, and the cell numbers of all the samples were more than 3,200. The mean transcripts per kilobase million (TPM) value of one gene was calculated, and the log2(TPM+1) was used as the expression value of the tumor cells in each sample. Given that too less tumor cells could not reflect the characteristics of the tumor, we remove two samples whose tumor cells were less than 50, and seven samples of stage IA LUAD were included for analysis.

### Construction and Validation of the MCPI

Based on the results of the multivariable Cox analysis in the all cohorts, age, stage, and MRGPI score were significantly associated with OS. Age, stage, and MRGPI score were integrated to composite a metabolism-clinical prognostic index (MCPI) by applying Cox proportional hazards regression in the meta-training cohort: MCPI score= *age × coefficient* + *stage × coefficient* + *MRGPI × coefficient*. The prognostic accuracy of MRGPI was estimated using the concordance index (C-index), which range from 0 to 1.0 (*survcomp* R package). As we mentioned above, the optimal cutoff value of MCPI score was determined by the *surv_cutpoint* function in the meta-training cohort. The predictive value of MCPI for OS was evaluated in the meta-testing and three independent validation cohorts.

### Statistical Analysis

All statistical analyses were conducted using R software (version 3.6.2). Pearson correlation analysis was performed to determine the correlation between two variables. The Kaplan–Meier method was used to generate survival curves, and significance of differences was compared using the log-rank test. All statistical tests were two-sided, and P values less than 0.05 were considered statistically significant.

## Results

### Patient Characteristics of Included Cohorts

Totally, 2,614 patients with stage I–II LUAD (**Table 1**) and 288 heathy donors were included for analysis. The median age ranged from 62 to 70 in all cohorts, and the proportion of female were larger than male. Most patients (>48.2%) had smoking history, and the patients with stage I LUAD accounted for the major proportion, except GSE68465, in which most patients did not had specific stage (stages I–II). In the meta-training, meta-testing, and GSE68465 cohorts, the median follow-up time was more than 50 months, and the death events were observed in more than 35% patients. However, the median follow-up time of the TCGA and GSE72094 was shorter, and the events of death were less than those of other cohorts.

**Table 1:** Clinical and pathologic features of patients in meta-training, meta-testing, and independent validation cohorts.

		Meta-training	Meta-testing	TCGA	GSE68465	GSE72094
Sample size, n		790	786	345	372	321
Age in years, median (IQR)		62 (56−69)	65 (58−72)	66 (59−72)	65 (58−72)	70 (64−77)
Sex, n (%)						
	Female	429 (54.3)	423 (53.8)	194 (56.2)	188 (50.5)	174 (54.2)
	Male	361 (45.7)	363 (46.2)	151 (43.8)	184 (49.5)	147 (45.8)
Smoking history, n (%)						
	Yes	381 (48.2)	398 (50.6)	288 (83.5)	257 (69.1)	244 (76.0)
	No	216 (27.3)	190 (24.2)	49 (14.2)	41 (11.0)	27 (8.4)
	Unknown	193 (24.4)	198 (25.1)	8 (2.3)	74 (19.9)	50 (15.6)
Stage, n (%)						
Stage I		625 (79.1)	601 (76.5)	237 (68.7)	115 (30.9)	254 (79.1)
	IA	278 (35.2)	221 (28.1)	117 (33.9)	115 (30.9)	150 (46.6)
	IB	260 (32.9)	264 (33.6)	115 (33.3)	–	99 (30.8)
	IA/B	87 (11.0)	116 (14.7)	5 (1.4)	–	5 (1.6)
Stage II		155 (19.6)	185 (23.5)	108 (31.3)	95 (25.5)	67 (20.9)
	IIA	21 (2.6)	42 (5.3)	47 (13.6)	–	18 (5.6)
	IIB	72 (9.1)	98 (12.5)	59 (17.1)	95 (25.5)	49 (15.3)
	IIA/B	62 (7.8)	45 (5.7)	2 (0.6)	–	–
Stages I−II		10 (1.3)	–	–	162 (43.5)	–
Follow-up in months, median (IQR)		56 (33−78)	50 (29−72)	19 (12−30)	52 (29−76)	27 (20−34)
No of death, n (%)		279 (35.3)	285 (36.3)	98 (28.4)	175 (47.0)	77 (24.0)

IRQ, interquartile range.

### Construction of the MRGPI

After pairwise coupling of the 690 GRPs, 237,705 MRGPs were constructed, and the corresponding scores were generated. We removed 205,031 MRGPs with CV <0.15 in all datasets and 210,771 MRGPs with CV >0.15 in the normal dataset. Between the remaining 32,674 MRGPs in the tumor cohorts and 26,934 MRGPs in the normal cohort, 856 MRGPs were overlapped. The association of the 856 MRGPs with OS was assessed in the meta-training cohort, resulting in 495 prognostic MRGPs. Finally, the LASSO Cox regression model selected 12 MRGPs in the meta-training cohort (**Supplementary Figure S2A**). Based on the 12 MRGPs that consisted of 20 MRGs, the MRGPI for each patient was constructed (**Table 2**). The optimal cutoff point (−0.261) obtained from the *surv_cutpoint* function served as the cutoff to assign patients into high- and low-risk groups (**Supplementary Figure S2B**). The Kaplan–Meier curve showed the patients in the high-risk group presented with a significantly worse OS in the meta-training cohort [hazard ratio (HR): 3.584, 95% confidence interval (CI): 2.755–4.663, P < 0.001, **Supplementary Figure S2C**]. Univariable Cox analysis indicated that MRGPI (both as continuous and binary form) was a prognostic factor for OS, and multivariable Cox analysis confirmed that MRGPI (as binary form) was independently associated with OS (**Figure 3** and **Supplementary Table S2**). The C-index of the MRGPI in the meta-training cohort was 0.701 (95% CI: 0.672–0.730).

**Table 2:** Model information about MRGPI.

MRGP	MRG 1	Full name	Function	MRG 2	Full name	Function	Coefficient
1	ALDH3A2	Aldehyde Dehydrogenase 3 Family Member A2	Catalyzing the oxidation of medium and long chain aliphatic aldehydes to fatty acids	GPX3	Glutathione Peroxidase 3	Catalyzing the reduction of hydrogen peroxide, lipid peroxides and organic hydroperoxide, by glutathione	−0.0049472424
2	AOC3	Amine Oxidase Copper Containing 3	Having semicarbazide-sensitive monoamine oxidase activity	CYP4F2	Cytochrome P450 Family 4 Subfamily F Member 2	Catalyzing many reactions involved in drug metabolism and synthesis of cholesterol, steroids and other lipids	−0.0223604279
3	DCTD	Deoxycytidylate Deaminase	Catalyzing the deamination of dCMP to dUMP, the nucleotide substrate for thymidylate synthase	B3GNT3	Beta-1,3-N-Acetylglucosaminyltransferase 3	Synthesis of poly-N-acetyllactosamine	−0.1552699047
4	GMPR	Guanosine Monophosphate Reductase	Catalyzing the irreversible NADPH-dependent deamination of GMP to IMP	CA5A	Carbonic Anhydrase 5A	Catalyzing the reversible hydration of carbon dioxide	−0.0076013442
5	B3GNT3	Beta-1,3-N-Acetylglucosaminyltransferase 3	Synthesis of poly-N-acetyllactosamine	HYAL2	Hyaluronidase 2	Hydrolyzing high molecular weight hyaluronic acid to produce an intermediate-sized product	0.0115559858
6	B3GNT3	Beta-1,3-N-Acetylglucosaminyltransferase 3	Synthesis of poly-N-acetyllactosamine	IMPDH1	Inosine Monophosphate Dehydrogenase 1	Catalyzing the conversion of IMP to XMP	0.0051310730
7	B3GNT3	Beta-1,3-N-Acetylglucosaminyltransferase 3	Synthesis of poly-N-acetyllactosamine	FPGS	Folylpolyglutamate Synthase	Catalyzing conversion of folates to polyglutamate derivatives	0.0328202856
8	SORD	Sorbitol Dehydrogenase	Catalyzing the reversible NAD(+)-dependent oxidation of various sugar alcohols	HEXA	Hexosaminidase Subunit Alpha	Degradation of GM2 gangliosides, and a variety of other molecules containing terminal N-acetyl hexosamines	0.0529668933
9	RPIA	Ribose 5-Phosphate Isomerase A	Catalyzing the reversible conversion between ribose-5-phosphate and ribulose-5-phosphate	NDUFAB1	NADH : Ubiquinone Oxidoreductase Subunit AB1	Carrier of the growing fatty acid chain in fatty acid biosynthesis	−0.0795029873
10	ALPI	Alkaline Phosphatase, Intestinal	Involving in folate biosynthesis	LCAT	Lecithin-Cholesterol Acyltransferase	Central enzyme in the extracellular metabolism of plasma lipoproteins	0.0012509917
11	ADH1C	Alcohol Dehydrogenase 1C	Gamma subunit of class I alcohol dehydrogenase that catalyzes ethanol oxidation to acetaldehyde	MAN2C1	Mannosidase Alpha Class 2C Member 1	Cleaving alpha 1,2-, alpha 1,3-, and alpha 1,6-linked mannose residues from glycoproteins	−0.0454175427
12	PFKFB4	6-Phosphofructo-2-Kinase/Fructose-2,6-Biphosphatase 4	Synthesis and degradation of fructose 2,6-bisphosphate	MAN2C1	Mannosidase Alpha Class 2C Member 1	Cleaving alpha 1,2-, alpha 1,3-, and alpha 1,6-linked mannose residues from glycoproteins	0.0756289035

dCMP, deoxycytidylic monophosphate; dUMP, deoxyuridine monophosphate; GMP, guanine monophosphate; IMP, inosine monophosphate; MRG, metabolism-related gene; MRGP, metabolism-related gene pair; NAD, nicotinamide adenine dinucleotide; NADPH, nicotinamide adenine dinucleotide phosphate; XMP, xanthosine monophosphate.

### Validation of the MRGPI in Multiple Independent Cohorts

**Figure 2:**
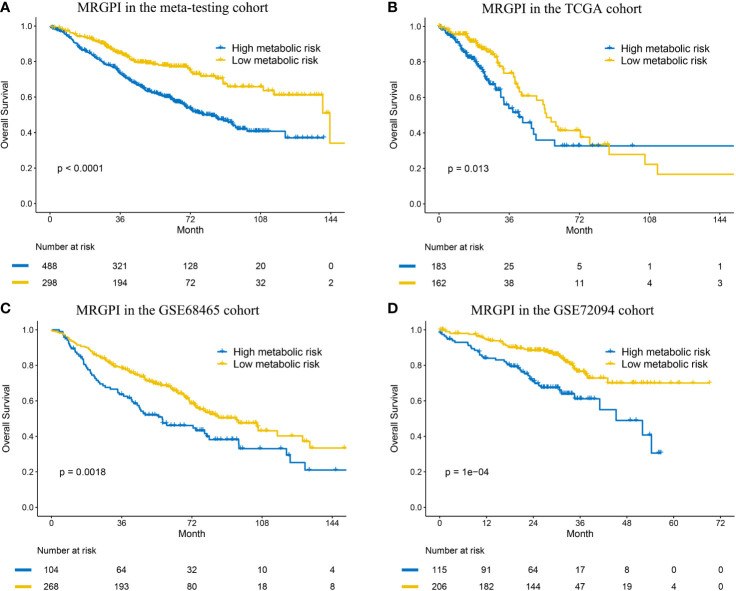
Kaplan–Meier curves of overall survival in the meta-testing **(A)**, TCGA **(B)** and GSE68465 **(C)** and GSE72094 **(D)** cohort.

**Figure 3:**
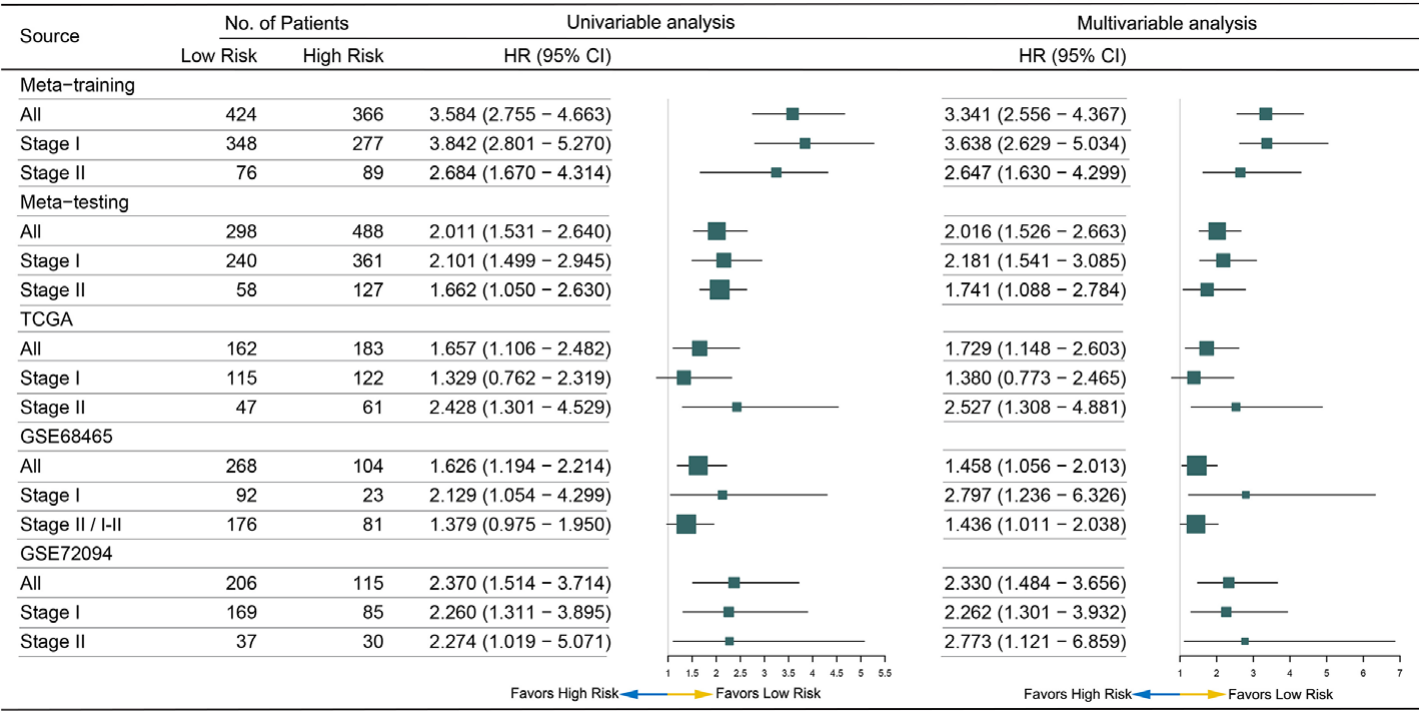
Forest plot for the hazard ratios (HRs) of high *vs* low metabolism-related gene pair index (MRGPI) risk groups.

To determine whether the MRGPI was robust, the performance of the MRGPI was assessed in the meta-testing and three independent cohorts. Consistent with the outcomes of the meta-training cohort, the MRGPI significantly stratified patients into low- *vs* high-risk groups in terms of OS. The patients in the high-risk group had significantly worse OS in the meta-testing (HR: 2.011, 95% CI: 1.531–2.640, P < 0.001, **Figure 2A**), TCGA (HR: 1.657, 95% CI: 1.106–2.482, P = 0.013, **Figure 2B**), GSE68465 (HR: 1.626, 95% CI: 1.194–2.214, P = 0.002, **Figure 2C**), and GSE72094 (HR: 2.370, 95% CI: 1.514–3.714, P < 0.001, **Figure 2D**) cohorts. The MRGPI (both as continuous and binary form) was a prognostic factor for OS in all the validation cohorts in the univariate Cox analysis, and it remained as an independent prognostic factor in multivariate analysis, after adjusting for age, gender, smoking history, and tumor stage (**Figure 3** and **Supplementary Table S2**). The C-index of the meta-testing, TCGA, GSE68465, GSE72094 cohort was 0.576 (95% CI: 0.541–0.612), 0.604 (95% CI: 0.535–0.673), 0.589 (95% CI: 0.543–0.634) and 0.645 (95% CI: 0.582–709), respectively.

### Subgroup Analysis of the MRGPI in Stage I Disease

In the patients with stage I disease, the MRGPI stratified patients in all cohorts into significantly different prognostic groups. The MRGPI remained highly prognostic for the meta-training (HR: 3.842, 95% CI: 2.801–5.270, P < 0.001), meta-testing (HR: 2.101, 95% CI: 1.499–2.945, P < 0.001), GSE68465 (HR: 2.129, 95% CI: 1.054–4.299, P = 0.031) and GSE72094 (HR: 2.260, 95% CI: 1.311–3.895, P = 0.003) cohort (**Supplementary Figures S2D** and **S3A–D**), and multivariable Cox analysis confirmed that MRGPI was independently associated with OS (**Figure 3** and **Supplementary Table S3**). However, the result was negative in the TCGA cohort, and the short follow-up time and less death events probably accounted for it.

**Figure 4:**
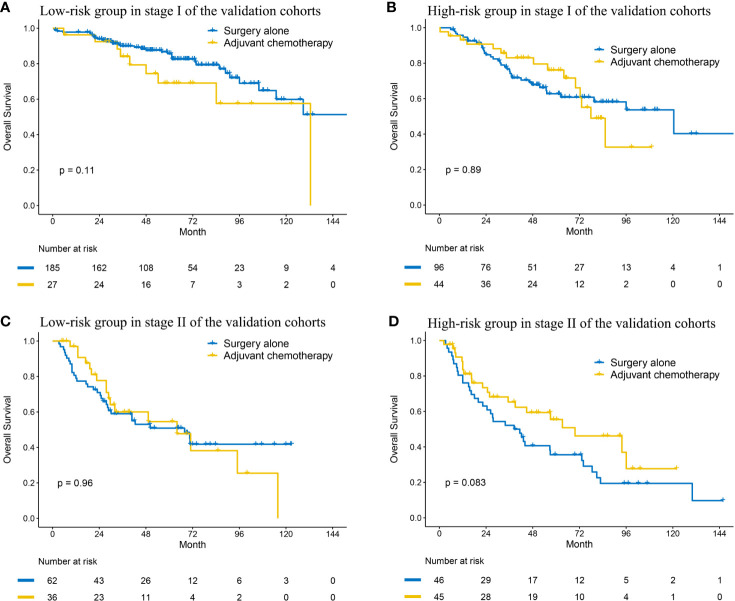
Kaplan–Meier curves of overall survival regarding adjuvant chemotherapy in patients with stage I **(A, B)** and stage II **(C, D)** disease at low and high risk in the validation cohort.

Given the prognosis differences between high- and low- risk patients, we analyzed the benefit of adjuvant chemotherapy (ACT) in the two groups. Of all the validation datasets, five datasets (OncoSG, GSE42127, GSE14814, TCGA, and GSE68465) recorded the information of ACT. Compared to surgery alone, ACT did not improve OS in the low-risk group (HR: 1.817, 95% CI: 0.871–3.791, P = 0.111; **Figure 4A**). We also did not observe that patients in the high-risk group could get OS benefit from ACT (HR: 0.959, 95% CI: 0.521–1.765, P = 0.893; **Figure 4B**), which indicated that ACT may be not suitable for the patients. To improve the prognosis, other adjuvant therapy regimens should be explored.

### Subgroup Analysis of the MRGPI in Stage II Disease

The MRGPI could also stratified patients in all cohorts into significantly different prognostic groups in the patients with stage II disease. The patients in the high-risk group had significantly worse OS in the meta-training (HR: 2.684, 95% CI: 1.670–4.314, P < 0.001), meta-testing (HR: 1.662, 95% CI: 1.050–2.630, P = 0.030), TCGA (HR: 2.428, 95% CI: 1.301–4.529, P < 0.001), and GSE72094 (HR: 2.274, 95% CI: 2.274, P = 0.045) cohort (**Supplementary Figures S2E** and **S4A–D**). The MRGPI remained an independent risk factor in multivariable analysis (**Figure 3** and **Supplementary Table S4**). A margin positive result (HR: 1.379, 95% CI: 0.975–1.950, P = 0.069) was observed in the GSE68465 cohort (including stages I–II, **Supplementary Figure S4C**); however, the result of multivariable analysis showed that the MRGPI was an independent risk factor (**Supplementary Table S4**).

Then, we also explored the effect of ACT in the two groups. The Kaplan–Meier curve indicated that ACT could not improve OS in the low-risk group (HR: 1.013, 95% CI: 0.561–1.829, P = 0.965; **Figure 4C**). In the high-risk group, although the result was negative (HR: 0.621, 95% CI: 0.360–1.070, P = 0.086; **Figure 4D**), the curves had an obvious tendency to separate and the small sample size probably accounted for it.

### Biological Phenotypes Associated With the MRGPI

**Figure 5:**
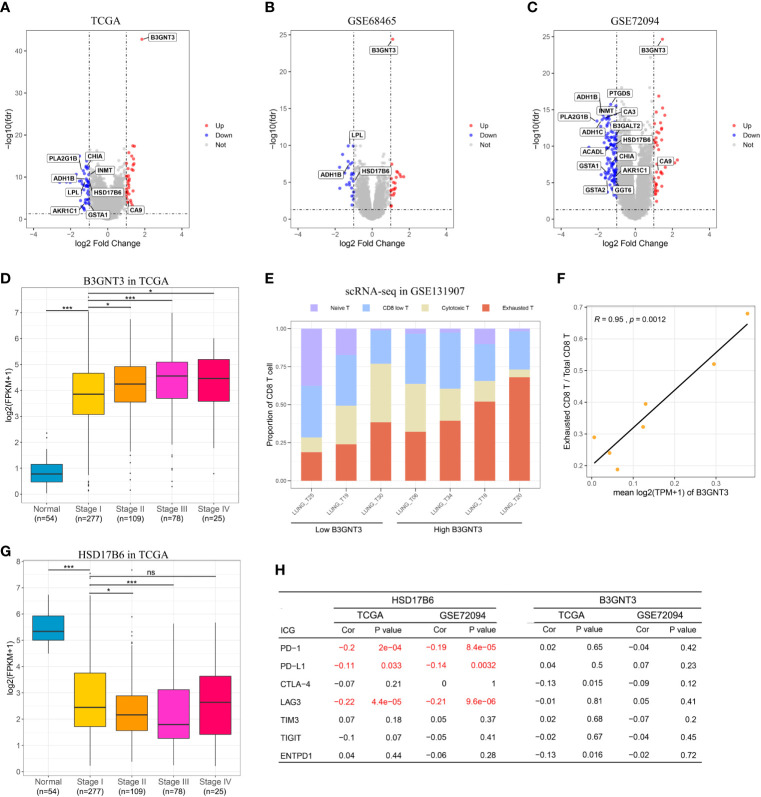
*In silico* analysis of the MRGPI. **(A–C)**: Volcano plot showing fold changes for genes differentially expressed between high- and low-risk patients in the TCGA, GSE68465, and GSE72094 cohort. **(D)** Boxplots of the expression level of B3GNT3 in the normal tissue and different tumor stages showing that upregulation of B3GNT correlated with tumor progression. **(E)** Proportion of different CD8^+^ T cell in each patient, and the patients were divided into low and high B3GNT3 group based on the median value. **(F)** The expression level of B3GNT3 was significantly associated with the proportion of the exhausted CD8^+^ T cell. **(G)** Boxplots of the expression level of HSD17B6 in the normal tissue and different tumor stages showing that down-regulation of HSD17B6 correlated with tumor progression. **(H)** Pearson’s correlation test between B3GNT3, HSD17B6, and immune checkpoint genes. ns, not significant (*P* > 0.05), **P < 0.01, ***P < 0.001.

Enrichment analysis of the 20 unique MRGs in the MRGPI identified two overrepresented biological processes (organic acid catabolic process and carboxylic acid catabolic process) in the gene ontology (**Supplementary Figure S5A**). To explore the potential survival mechanism related to the MRGPI, we analyzed the DEGs between the high and low-risk groups in the three independent validation cohorts, and we focused on the differentially expressed MRGs. Among the DEGs from the three cohorts, three MRGs (B3GNT3, ADH1B, and HSD17B6) were overlapped (**Figures 5A** **–C**), and their expression levels were significantly correlated with MRGPI (**Supplementary Figure S6**). The three MRGs had been reported to be associated with other cancers (17–19), but few studies reported their role in LUAD.

B3GNT3 was overexpressed in LUAD, and its expression level was positively associated with tumor stage (**Figure 5D**), which suggested that B3GNT3 played an important role in tumor carcinogenesis and prognosis. Previous study reported that N-linked glycosylation of PD-L1 that was catalyzed by B3GNT3 was required for physical contact between PD-L1 and PD-1 in triple-negative breast cancer, and then caused CD8^+^ T cell exhausted (18). We then explored whether there was a similar mechanism in LUAD. From the scRNA-seq result, we noticed that the expression level of B3GNT3 in tumor cell was positively correlated with the proportion of the exhausted CD8^+^ T cell (r = 0.95, P = 0.0012, **Figures 5E, F** **)**. However, the expression level of B3GNT3 was not correlated with immune checkpoint genes (ICGs) in the TCGA and GSE72094 cohorts (most ICGs were not available in the GSE68465 dataset), especially PD-1 and PD-L1 (**Figure 5H**). The results demonstrated that there may be the same mechanism of B3GNT3 in LUAD.

HSD17B6 was down-expressed in LUAD, and the expression level of HSD17B6 was negatively associated with tumor stage (**Figure 5G**). HSD17B6 could convert 3 alpha-adiol to dihydrotestosterone that was closely related to the development of many tumors (20). Lv et al. (17) reported that low expression of HSD17B6 correlated with multiple ICGs expression in hepatocellular carcinoma. In this study, we observed that the expression level of HSD17B6 was negatively correlated with PD-1 (r = −0.20 and P < 0.001 in TCGA, r = −0.19 and P < 0.001 in GSE72094), PD-L1 (r = −0.11 and P = 0.033 in TCGA, r = −0.14 and P = 0.003 in GSE72094), and LAG3 (r = −0.22 and P < 0.001 in TCGA, r = -0.21 and P < 0.001 in GSE72094) (**Figure 5H**), suggesting that low HSD17B6 expression potentially played an important role in mediating immune evasion. ADH1B was also down-expressed in LUAD (**Supplementary Figure S5B**); however, its expression level was not negatively correlated with ICGs as HSD17B6 (**Supplementary Figure S5C**), which suggested that there may be other mechanisms behind it.

Together, these results indicated that B3GNT3 and HSD17B6 may make synergic reaction in immune evasion, with HSD17B6 up-regulating PD-L1 and B3GNT3 stabilizing the PD-L/PD-L1 ligation. Immune checkpoint inhibitors, especially PD-1/PD-L1 anti-body may be a therapeutic choice. Combined with the results of ACT in LUAD at different stages and risks, we thought that patients at high risk may get survival benefit from PD-1/PD-L1 blockade (stage I) or combined with chemotherapy (stage II). Although PD-1/PD-L1 anti-body as neoadjuvant therapy has been used in early stage NSCLC in clinical trials recently (21–24), there are no transcriptomic data of the tumor before treatment at present, so the regimen we proposed could not be validated in this study.

### Integrated Prognostic Index by Combining the MRGPI With Clinical Factors

To further improve accuracy, we combined age, stage, and MRGPI score to fit a Cox proportional hazards regression model in the meta-training cohort and derived a MCPI: MCPI = age × 0.028 + stage × 0.312 + MRGPI × 1.726. The optimal cutoff value of the MCPI for stratifying patients was determined to be 2.007 (**Supplementary Figure S7A**). Improved estimation of OS was achieved by the binary form of MCPI compared with MRGPI (**Supplementary Figures S7B−F**), and the C-index for the meta-training, meta-testing, TCGA, GSE68465, GSE72094 cohort was 0.729 (95% CI 0.700–0.757), 0.648 (95% CI 0.613–0.682), 0.641 (95% CI 0.567–0.709), 0.665 (95% CI 0.634–0.709), and 0.666 (95% CI 0.602–0.731), respectively (**Supplementary Figure S7G**).

## Discussion

When diagnosed at early stages, LUAD could be effectively treated with surgical resection. However, the use of ACT for stage I LUAD in the setting of standard therapy remains controversial because several clinical trials fail to show a survival benefit among unselected patients, and the toxic effects of chemotherapy are inevitable (25). The strategy is to identify of the subset of patients at high risk for recurrence and death. A prognostic signature beyond the current staging system is desired to accurately identify the patients at high risk and to better guide adjuvant treatment (7). In this study, we developed a prognostic signature based on 12 MRGPs to predict prognosis of early stage LUAD and validated it in multiple independent cohorts across different platforms. The MRGPI was extremely robust in stratifying the patients into the low- and high-risk groups with different survival outcomes. Several models based on the expression value have already been reported to present with the ability for predicting prognosis in lung cancer (26–29). However, the models based on the absolute value of the expression level could not avoid the technical biases inherent across different platforms. The gene pairs signature proposed by Li et al. (6) is based on the relative value of gene expression level, which only refers to the pairwise comparison of the gene expression profile within a sample. Li et al. constructed a gene pair signature based on 25 immune-related gene pairs consisting of 40 immune-related genes in non-squamous lung cancer (6). Our prognostic signature was derived from MRGs in LUAD and MRGPI consisted of 12 gene pairs involving 20 MRGs. With less gene pairs, MRGPI performed comparable accuracy to Li and colleagues’ model in the TCGA (C-index: 0.60 *vs* 0.62) cohort.

After identifying the patients at different risks, we explored the benefit of ACT. Not surprisingly, ACT could not bring survival benefit in stage I LUAD at low risk. However, ACT also could not improve OS in stage I LUAD at high risk, suggesting that chemotherapy may be not suitable for the patients. For stage II LUAD, ACT may improve OS in the patients at high risk, which was in accordance with the clinical practice. However, we also noticed that the patients at low risk could not get survival benefit from ACT, suggesting that ACT should also be used selectively in a subset of patients with stage II LUAD. According to the NCCN guidelines, ACT should be performed in stage IIB LUAD with R0 resection, but it is alternative in stage IIA LUAD and just required for high-risk patients (4). Besides identifying high-risk patients with stage I LUAD, MRGPI could also identify a subset of patients in stage II who may be free from ACT. However, the sample size of ACT was small in this study, and more studies were needed to validate the results.

To explore potential therapeutic targets for the patients with poor prognosis based on the MRGPI, we performed DEG analysis using the three independent datasets. Three MRGs were identified, and B3GNT3 and HSD17B6 may make synergic reaction in immune evasion by the PD-1/PD-L1 pathway. Thus, PD-1/PD-L1 blockade was an optimal therapy regimen for the patients at high risk. Compared with conventional ACT, adjuvant immunotherapy could improve prognosis in resectable solid tumor (30, 31), and neoadjuvant therapy may get more survival benefit than adjuvant therapy (32). Recently, PD-1/PD-L1 anti-body as neoadjuvant therapy has been proved to be feasible in resectable lung cancer (21−24). Thus, the patients with stage I LUAD at high risk may be get survival benefit from PD-1/PD-L1 blockade. For the patients with stage II LUAD at high risk, both chemotherapy and PD-1/PD-L1 blockade may improve prognosis, so PD-1/PD-L1 anti-body plus chemotherapy as neoadjuvant therapy may be optimal. However, there are no transcriptomic data of the tumor before immunotherapy available at present to validate it. For the patients at low risk, surgery alone may be optimal, but the benefit of immunotherapy should also be explored in future studies.

There were some limitations in our study. First, some biases were inevitable because of the retrospective nature of this study. Second, the mutation status was not considered due to lack of information of most datasets. Since driver genes like EGFR and ALK mutation were common in LUAD, the benefit of targeted therapy in the patients at risk could not be evaluated, and adjuvant targeted therapy was proved to be better than ACT in clinical trials (33, 34). Third, as we mentioned above, the sample size of ACT was small, and more studies were needed to validate the results. Last, the therapy regimens we proposed were warranted to validate in clinical studies.

In conclusion, this study identified metabolism-related gene pair-based signature that can effectively predict survival outcomes of the patients with early stage LUAD. The patients at high risk may get survival benefit from PD-1/PD-L1 blockade (stage I) or combined with chemotherapy (stage II). Prospective studies are needed to further validate its analytical accuracy for estimating prognosis and test its clinical utility in individualized management of early stage LUAD.

## Data Availability Statement

The datasets presented in this study can be found in online repositories. The names of the repository/repositories and accession number(s) can be found in the article/**Supplementary Material**.

## Ethics Statement

The studies involving human participants were reviewed and approved by Tongji University. Written informed consent for participation was not required for this study in accordance with the national legislation and the institutional requirements.

## Author Contributions

Conception and design: PZ. Administrative support: GJ. Provision of study materials or patients: JH, HY, and LZ. Collection and assembly of data: JH, LS, and YY. Data analysis and interpretation: JH, LS, and HY. Manuscript writing: all authors. All authors contributed to the article and approved the submitted version.

## Funding

This work was supported by the National Key R&D Program of China (2019YFC1315803), National Natural Science Foundation of China (Grant No. 81972172), the Shanghai Municipal Health Commission (Grant No. 20174Y0111), the Shanghai Science and Technology Committee (Grant No. 19XD1423200, 18140903900, 201409001000), and Programs of Shanghai Pulmonary Hospital (No. fkcx1904).

## Conflict of Interest

The authors declare that the research was conducted in the absence of any commercial or financial relationships that could be construed as a potential conflict of interest.
