# Supplementary material for: Identification of an Individualized Metabolism Prognostic Signature and Related Therapy Regimens in Early Stage Lung Adenocarcinoma
Source: Front Oncol. 2021 Apr 28;11:650853. doi: 10.3389/fonc.2021.650853 (PMC8113858; doi:10.3389/fonc.2021.650853)
Supplement: Supplementary file 1 [file DataSheet_1.docx]

**Supplementary materials**

**Supplementary Table**

Table S1. Details about the datasets used in this study

|  | Name/  Accession No. | Platform | No. of Adenocarcinoma |
| --- | --- | --- | --- |
| Meta-training dataset | GSE31210 | Affymetrix Human Genome U133 Plus 2.0 Array | 226 |
|  | GSE41271 | Illumina HumanWG-6 v3.0 expression beadchip | 128 |
|  | GSE83227 | Affymetrix Human Genome U95 Version 2 Array | 99 |
|  | GSE37745 | Affymetrix Human Genome U133 Plus 2.0 Array | 89 |
|  | GSE30219 | Affymetrix Human Genome U133 Plus 2.0 Array | 84 |
|  | GSE11969 | Agilent Homo sapiens 21.6K custom array | 65 |
|  | E-MTAB-923 | Affymetrix Human Genome U133 Plus 2.0 Array | 63 |
|  | GSE87340 | Illumina HiSeq 2000 (Homo sapiens) | 20 |
|  | GSE31546 | Affymetrix Human Genome U133 Plus 2.0 Array | 16 |
| Meta-testing dataset | OncoSG | Illumina HiSeq 2000 | 132 |
|  | GSE50081 | Affymetrix Human Genome U133 Plus 2.0 Array | 127 |
|  | GSE42127 | Illumina HumanWG-6 v3.0 expression beadchip | 111 |
|  | GSE13213 | Agilent-014850 Whole Human Genome Microarray 4x44K G4112F | 92 |
|  | GSE26939 | Agilent-UNC-custom-4X44K | 81 |
|  | GSE81089 | Illumina HiSeq 2500 (Homo sapiens) | 81 |
|  | GSE14814 | Affymetrix Human Genome U133A Array | 71 |
|  | GSE29016 | Illumina HumanHT-12 V3.0 expression beadchip | 34 |
|  | GSE63459 | Illumina HumanRef-8 v3.0 expression beadchip | 32 |
|  | GSE31547 | Affymetrix Human Genome U133A Array | 25 |
| Independent dataset | TCGA | Illumina HiSeq | 345 |
|  | GSE68465 | Affymetrix Human Genome U133A Array | 372 |
|  | GSE72094 | Rosetta/Merck Human RSTA Custom Affymetrix 2.0 microarray | 321 |

Table S2. Univariable and multivariable analyses of prognostic factors in meta-training, meta-testing, and independent validation cohorts

| Datasets | Variable | Univariable | | Multivariable | |
| --- | --- | --- | --- | --- | --- |
|  |  | HR (95% CI) | P Value | HR (95% CI) | P Value |
| Meta-training | Age | 1.035 (1.022 - 1.049) | 1.83×10^-7^ | 1.030 (1.017 - 1.044) | 4.16×10^-6^ |
|  | Gender (male vs female) | 1.338 (1.056 - 1.696) | 1.57×10^-2^ | 1.238 (0.973 - 1.575) | 0.082 |
|  | Smoking (Yes vs no/unknown) | 1.058 (0.832 - 1.345) | 0.645 | 0.952 (0.743 - 1.220) | 0.701 |
|  | Stage | 1.462 (1.310 - 1.630) | 9.78×10^-12^ | 1.358 (1.213 - 1.520) | 1.06×10^-7^ |
|  | MRGPI | 6.406 (4.556 - 9.008) | < 2×10^-16^ | - | - |
|  | Metabolic risk (high vs low) | 3.584 (2.755 - 4.663) | < 2×10^-16^ | 3.341 (2.556 - 4.367) | < 2×10^-16^ |
| Meta-testing | Age | 1.025 (1.013 - 1.037) | 2.76×10^-5^ | 1.027 (1.014 - 1.039) | 1.78×10^-5^ |
|  | Gender (male vs female) | 1.592 (1.260 - 2.010) | 0.016 | 1.377 (1.082 - 1.752) | 9.24×10^-3^ |
|  | Smoking (Yes vs no/unk) | 1.305 (1.024 - 1.663) | 9.60×10^-5^ | 1.305 (1.024 - 1.663) | 0.031 |
|  | Stage | 1.436 (1.289 - 1.601) | 5.84×10^-11^ | 1.394 (1.248 - 1.556) | 3.63×10^-9^ |
|  | MRGPI | 2.413 (1.652 - 3.526) | 5.29×10^-6^ |  |  |
|  | Metabolic risk (high vs low) | 2.011 (1.531 - 2.640) | 4.95×10^-7^ | 2.016 (1.526 - 2.663) | 8.00×10^-7^ |
| TCGA | Age | 1.026 (1.004 - 1.049) | 0.021 | 1.037 (1.012 - 1.062) | 2.84×10^-3^ |
|  | Gender (male vs female) | 1.214 (0.816 - 1.807) | 0.338 | 1.102 (0.731 - 1.659) | 0.642 |
|  | Smoking (Yes vs no/unknown) | 0.992 (0.594 - 1.656) | 0.976 | 1.398 (0.807 - 2.420) | 0.231 |
|  | Stage | 1.426 (1.196 - 1.700) | 7.59×10^-5^ | 1.483 (1.232 - 1.783) | 2.92×10^-5^ |
|  | MRGPI | 3.061 (1.618 - 5.791) | 5.85×10^-4^ |  |  |
|  | Metabolic risk (high vs low) | 1.657 (1.106 - 2.482) | 0.014 | 1.729 (1.148 - 2.603) | 8.76×10^-3^ |
| GSE68465 | Age | 1.032 (1.017 - 1.049) | 5.58×10^-5^ | 1.038 (1.021 - 1.055) | 9.37×10^-6^ |
|  | Gender (male vs female) | 1.417 (1.049 - 1.915) | 0.023 | 1.281 (0.946 - 1.734) | 0.109 |
|  | Smoking (Yes vs no/unknown) | 1.038 (0.964 - 1.430) | 0.821 | 1.189 (0.853 - 1.657) | 0.307 |
|  | Stage | 1.461 (1.273 - 1.676) | 6.32×10^-8^ | 1.417 (1.229 - 1.635) | 1.69×10^-6^ |
|  | MRGPI | 2.448 (1.276 - 4.699) | 7.11×10^-3^ | - | - |
|  | Metabolic risk (high vs low) | 1.626 (1.194 - 2.214) | 2.05×10^-3^ | 1.458 (1.056 - 2.013) | 0.022 |
| GSE72094 | Age | 1.007 (0.984 - 1.030) | 0.547 | 1.008 (0.985 - 1.030) | 0.504 |
|  | Gender (male vs female) | 1.475 (0.942 - 2.311) | 0.089 | 1.641 (1.024 - 2.631) | 0.039 |
|  | Smoking (Yes vs no/unknown) | 0.673 (0.406 - 1.114) | 0.123 | 0.612 (0.360 - 1.040) | 0.070 |
|  | Stage | 1.298 (1.072 - 1.572) | 7.67×10^-3^ | 1.274 (1.046 - 1.552) | 0.016 |
|  | MRGPI | 5.385 (2.510 - 11.55) | 1.53×10^-5^ | - | - |
|  | Metabolic risk (high vs low) | 2.370 (1.514 - 3.714) | 1.61×10^-4^ | 2.330 (1.484 - 3.656) | 2.34×10^-4^ |

Table S3. Univariable and multivariable analyses of prognostic factors for stage I disease in meta-training, meta-testing, and independent validation cohorts.

| Datasets | Variable | Univariable | | Multivariable | |
| --- | --- | --- | --- | --- | --- |
|  |  | HR (95% CI) | P Value | HR (95% CI) | P Value |
| Meta-training | Age | 1.043 (1.027 - 1.060) | 1.74×10^-7^ | 1.037 (1.021 - 1.053) | 5.64×10^-6^ |
|  | Gender (male vs female) | 1.383 (1.042 - 1.835) | 2.49×10^-2^ | 1.261 (0.945 - 1.684) | 0.115 |
|  | Smoking (Yes vs no/unknown) | 1.053 (0.788 - 1.406) | 0.728 | 0.960 (0.714 - 1.291) | 0.786 |
|  | Stage | 2.259 (1.638 - 3.116) | 6.7×10^-7^ | 1.701 (1.225 - 2.363) | 1.52×10^-3^ |
|  | MRGPI | 6.858 (4.580 - 10.27) | < 2×10^-16^ | - | - |
|  | Metabolic risk (high vs low) | 3.842 (2.801 - 5.270) | < 2×10^-16^ | 3.638 (2.629 - 5.034) | 6.50×10^-15^ |
| Meta-testing | Age | 1.025 (1.010 - 1.040) | 6.93×10^-4^ | 1.027 (1.012 - 1.042) | 3.17×10^-4^ |
|  | Gender (male vs female) | 1.472 (1.103 - 1.965) | 8.63×10^-3^ | 1.288 (0.950 - 1.745) | 0.103 |
|  | Smoking (Yes vs no/unk) | 1.332 (0.990 - 1.793) | 0.058 | 1.272 (0.935 - 1.729) | 0.125 |
|  | Stage | 1.510 (1.084 - 2.104) | 0.015 | 1.396 (0.997 - 1.956) | 0.052 |
|  | MRGPI | 2.297 (1.443 - 3.655) | 4.52×10^-4^ | - | - |
|  | Metabolic risk (high vs low) | 2.101 (1.499 - 2.945) | 1.63×10^-5^ | 2.181 (1.541 - 3.085) | 1.06×10^-5^ |
| TCGA | Age | 1.043 (1.010 - 1.076) | 9.63×10^-3^ | 1.045 (1.012 - 1.079) | 6.44×10^-3^ |
|  | Gender (male vs female) | 0.758 (1.425 - 1.349) | 0.346 | 0.732 (0.407 - 1.318) | 0.299 |
|  | Smoking (Yes vs no/unknown) | 0.888 (0.567 - 1.743) | 0.984 | 1.055 (0.481 - 2.315) | 0.893 |
|  | Stage | 0.994 (0.567 - 1.743) | 0.984 | 0.899 (0.507 - 1.595) | 0.715 |
|  | MRGPI | 2.694 (1.170 - 6.204) | 0.020 | - | - |
|  | Metabolic risk (high vs low) | 1.329 (0.762 - 2.319) | 0.316 | 1.380 (0.773 - 2.465) | 0.275 |
| GSE68465 | Age | 1.040 (1.007 - 1.075) | 0.017 | 1.057 (1.017 - 1.099) | 5.22×10^-3^ |
|  | Gender (male vs female) | 2.209 (1.162 - 4.200) | 0.016 | 2.328 (1.200 - 4.517) | 0.012 |
|  | Smoking (Yes vs no/unknown) | 0.598 (0.314 - 1.138) | 0.117 | 0.794 (0.397 - 1.588) | 0.514 |
|  | Stage | - | - | - | - |
|  | MRGPI | 3.103 (0.789 - 12.21) | 0.105 | - | - |
|  | Metabolic risk (high vs low) | 2.129 (1.054 - 4.299) | 0.035 | 2.797 (1.236 - 6.326) | 0.013 |
| GSE72094 | Age | 1.006 (0.978 - 1.035) | 0.664 | 1.000 (0.972 - 1.029) | 0.978 |
|  | Gender (male vs female) | 1.850 (1.063 - 3.221) | 0.029 | 2.226 (1.191 - 4.162) | 0.012 |
|  | Smoking (Yes vs no/unknown) | 0.780 (0.409 - 1.491) | 0.453 | 0.582 (0.282 - 1.202) | 0.143 |
|  | Stage | 0.951 (0.543 - 1.668) | 0.862 | 0.787 (0.445 - 1.393) | 0.412 |
|  | MRGPI | 4.760 (1.911 - 11.86) | 8.08×10^-4^ | - | - |
|  | Metabolic risk (high vs low) | 2.260 (1.311 - 3.895) | 3.35×10^-3^ | 2.262 (1.301 - 3.932) | 3.81×10^-3^ |

Table S4. Univariable and multivariable analyses of prognostic factors for stage II disease in meta-training, meta-testing, and independent validation cohorts.

| Datasets | Variable | Univariable | | Multivariable | |
| --- | --- | --- | --- | --- | --- |
|  |  | HR (95% CI) | P Value | HR (95% CI) | P Value |
| Meta-training | Age | 1.023 (0.999 - 1.046) | 0.051 | 1.016 (0.994 - 1.040) | 0.159 |
|  | Gender (male vs female) | 1.030 (0.667 - 1.590) | 0.893 | 1.086 (0.678 - 1.740) | 0.731 |
|  | Smoking (Yes vs no/unknown) | 0.900 (0.583 - 1.389) | 0.634 | 0.984 (0.611 - 1.585) | 0.947 |
|  | Stage | 1.032 (0.649 - 1.640) | 0.895 | 0.959 (0.605 - 1.520) | 0.859 |
|  | MRGPI | 4.867 (2.501 - 9.469) | 3.17×10^-6^ | - | - |
|  | Metabolic risk (high vs low) | 2.684 (1.670 - 4.314) | 4.53×10^-5^ | 2.647 (1.630 - 4.299) | 8.3×10^-5^ |
| Meta-testing | Age | 1.024 (1.003 - 1.046) | 0.027 | 1.025 (1.005 - 1.047) | 0.030 |
|  | Gender (male vs female) | 1.648 (1.102 - 2.466) | 0.015 | 1.567 (1.042 - 2.357) | 0.031 |
|  | Smoking (Yes vs no/unk) | 1.577 (1.057 - 2.352) | 0.026 | 1.415 (0.937 - 2.137) | 0.099 |
|  | Stage | 1.439 (0.875 - 2.366) | 0.152 | 1.192 (0.723 - 1.966) | 0.491 |
|  | MRGPI | 2.013 (1.043 - 3.885) | 0.037 | - | - |
|  | Metabolic risk (high vs low) | 1.662 (1.050 - 2.630) | 0.030 | 1.741 (1.088 - 2.784) | 0.021 |
| TCGA | Age | 1.016 (0.983 - 1.051) | 0.342 | 1.032 (0.996 - 1.069) | 0.085 |
|  | Gender (male vs female) | 1.967 (1.072 - 3.610) | 0.029 | 1.778 (0.917 - 3.448) | 0.088 |
|  | Smoking (Yes vs no/unknown) | 1.430 (0.700 - 2.923) | 0.327 | 1.297 (0.598 - 2.816) | 0.510 |
|  | Stage | 0.831 (0.452 - 1.528) | 0.551 | 0.628 (0.331 - 1.192) | 0.155 |
|  | MRGPI | 3.906 (1.259 - 12.120) | 0.018 | - | - |
|  | Metabolic risk (high vs low) | 2.428 (1.301 - 4.529) | 5.32×10^-3^ | 2.527 (1.308 - 4.881) | 5.78×10^-3^ |
| GSE68465 (including I-II) | Age | 1.033 (1.014 - 1.052) | 4.29×10^-4^ | 1.034 (1.015 - 1.052) | 3.21×10^-4^ |
|  | Gender (male vs female) | 1.122 (0.797 - 1.578) | 0.510 | 1.130 (0.802 - 1.591) | 0.484 |
|  | Smoking (Yes vs no/unknown) | 1.198 (0.819 - 1.750) | 0.352 | 1.339 (0.910 - 1.969) | 0.138 |
|  | Stage | - | - | - | - |
|  | MRGPI | 1.670 (0.769 - 3.625) | 0.195 | - | - |
|  | Metabolic risk (high vs low) | 1.379 (0.975 - 1.950) | 0.069 | 1.436 (1.011 - 2.038) | 0.043 |
| GSE72094 | Age | 1.011 (0.972 - 1.051) | 0.581 | 1.027 (0.986 - 1.070) | 0.201 |
|  | Gender (male vs female) | 0.800 (0.361 - 1.773) | 0.583 | 0.931 (0.410 - 2.112) | 0.864 |
|  | Smoking (Yes vs no/unknown) | 0.619 (0.270 - 1.417) | 0.256 | 0.622 (0.251 - 1.541) | 0.305 |
|  | Stage | 0.941 (0.372 - 2.76) | 0.897 | 0.795 (0.272 - 2.325) | 0.675 |
|  | MRGPI | 5.077 (1.226 - 21.02) | 0.025 | - | - |
|  | Metabolic risk (high vs low) | 2.274 (1.019 - 5.071) | 0.045 | 2.773 (1.121 - 6.859) | 0.027 |

**Supplementary Figure**


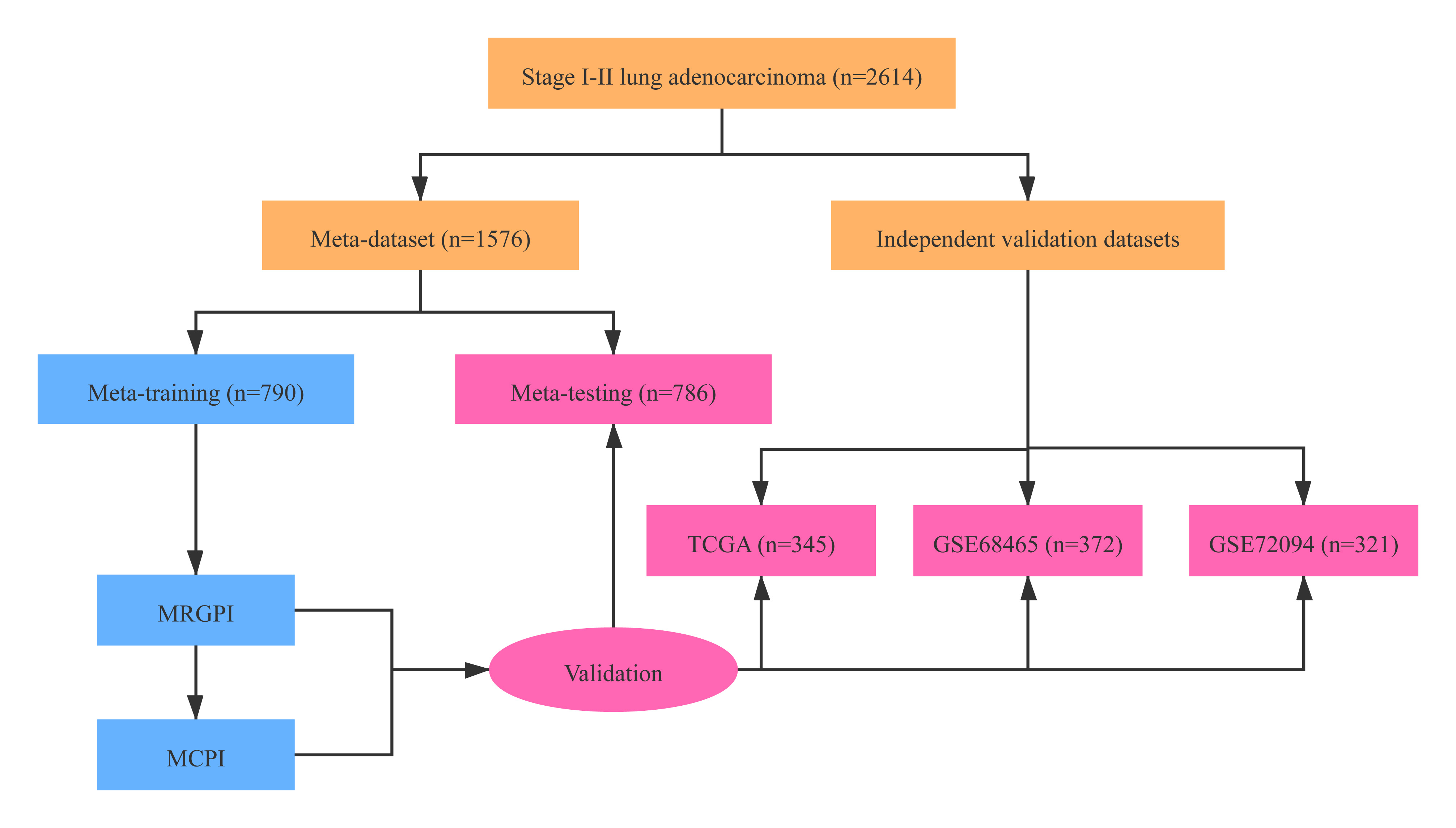


**Figure S1.** Overview of the construction and validation of metabolism and composite metabolism/clinical signatures. Totally, 22 datasets were included in this study. The 3 largest individual datasets were used for independent validation, while the remaining 19 datasets were merged to a meta-dataset. The meta-dataset was randomly divided into meta-training and meta-testing datasets. The meta-training dataset was used to build a metabolism prognostic signature (MRGPI). Age, stage and MRGPI were used to construct the composite metabolism clinical prognostic signature (MCPI). Both MRGPI and MCPI were evaluated on the meta-testing, TCGA, GSE68465 and GSE72094 datasets.


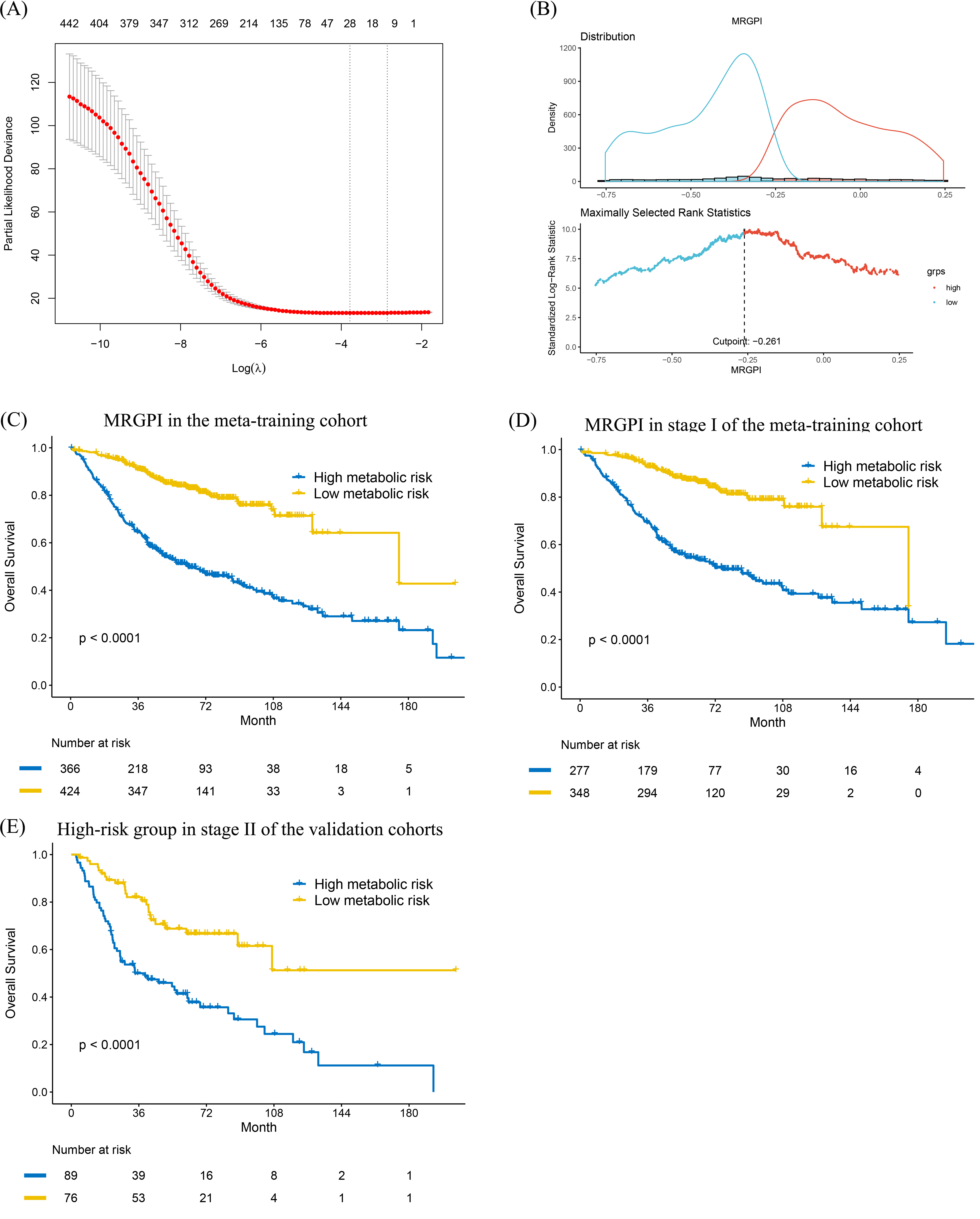


**Figure S2.** (A) Partial likelihood deviance of different numbers of variables revealed by the LASSO regression model. The red dots represent the partial likelihood deviance values, and the grey lines represent the standard error (SE), and the two vertical dotted lines on the left and right, respectively, represent optimal values by minimum criteria and 1-SE criteria. (B) Optimal cutoff value of MRGPI selected by the maximally selected rank statistics. The MRGPI corresponding to the max statistic is the optimal cutoff value. Kaplan-Meier curves of overall survival in the meta-training cohort (C), and in stage I (D) and stage II (E) disease.


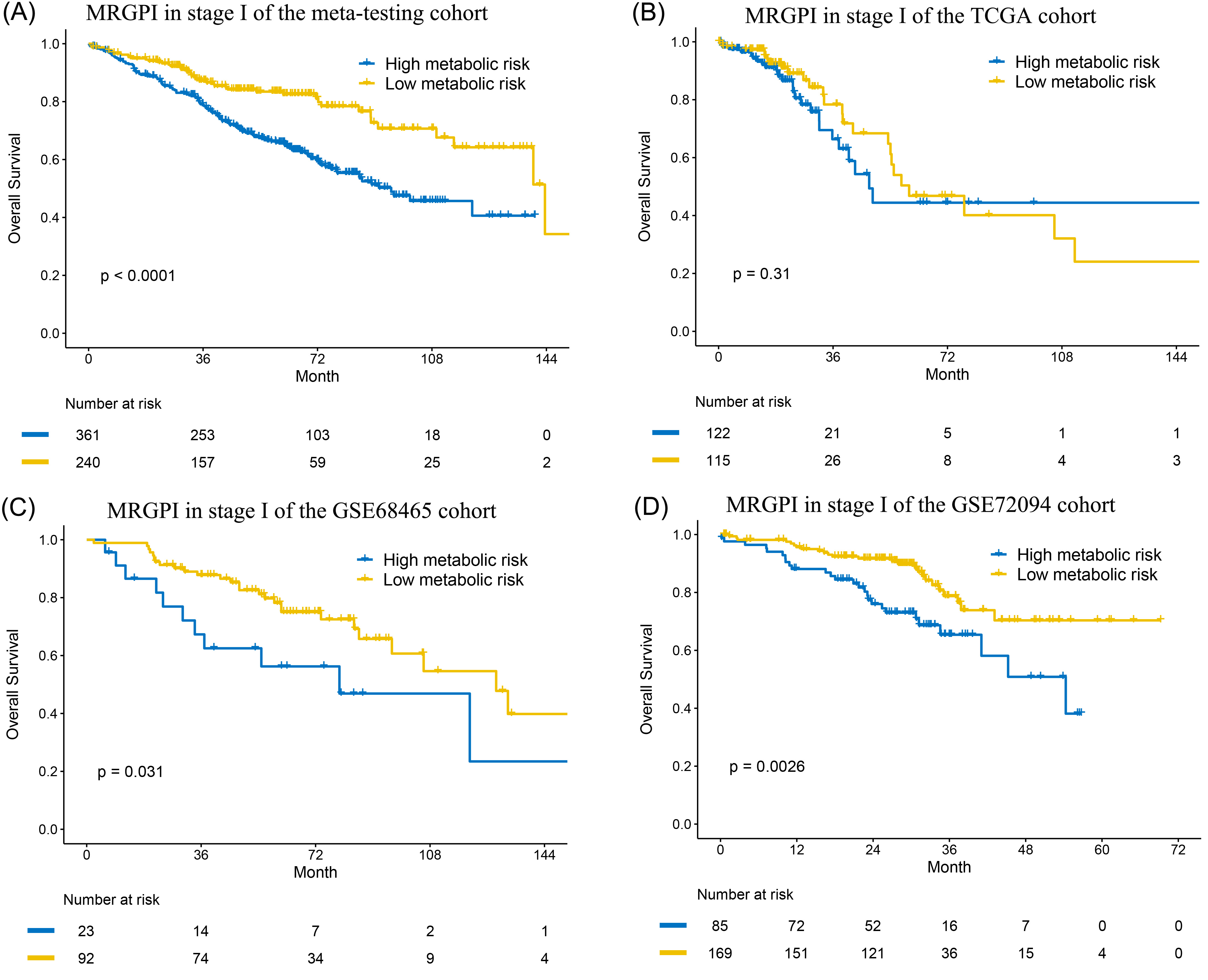


**Figure S3.** Kaplan-Meier curves of overall survival among the patients in stage I disease in the meta-testing (A), TCGA (B) and GSE68465 (C) and GSE72094 (D) cohort.


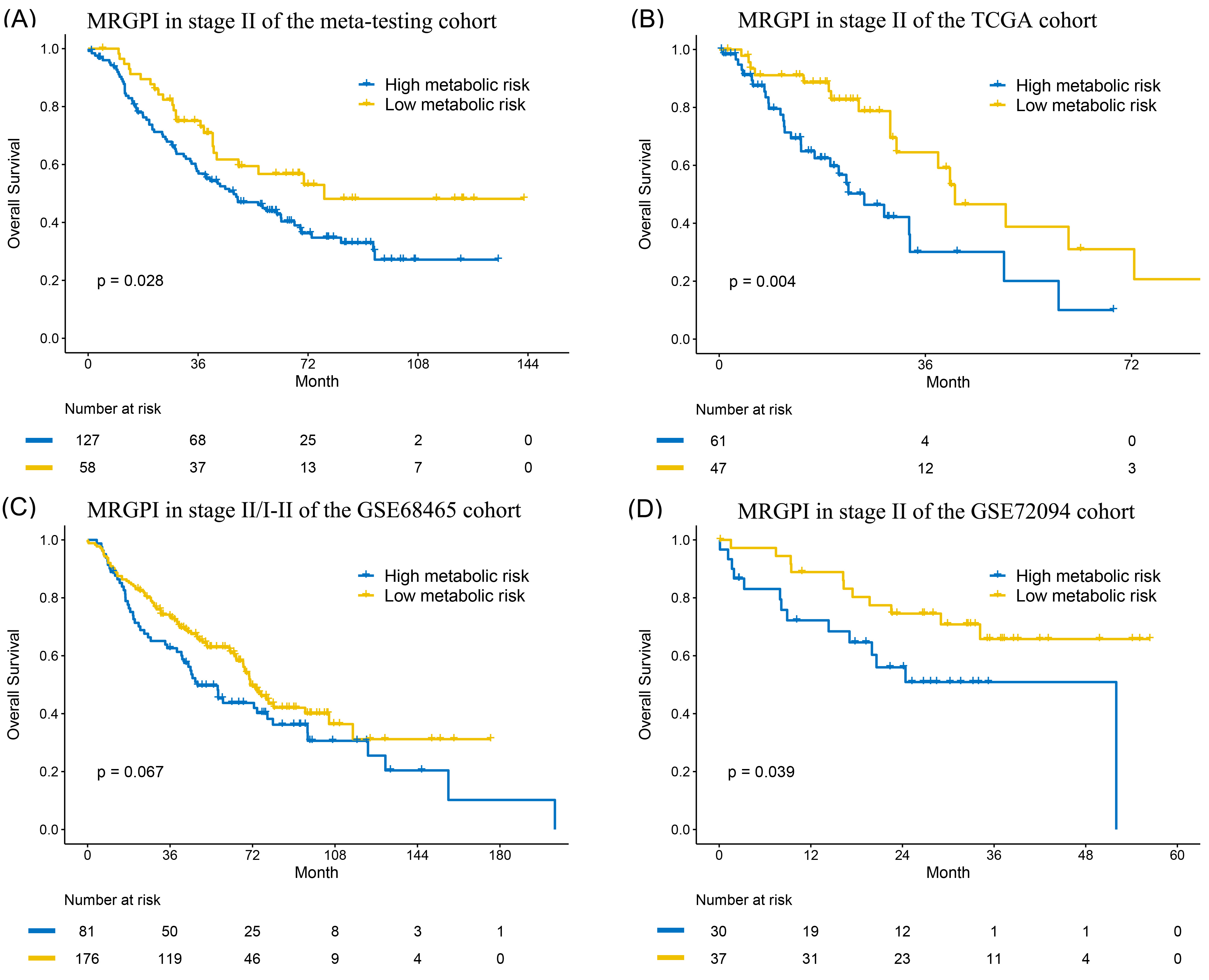


**Figure S4.** Kaplan-Meier curves of overall survival among the patients in stage II disease in the meta-testing (A), TCGA (B) and GSE68465 (C) and GSE72094 (D) cohort.


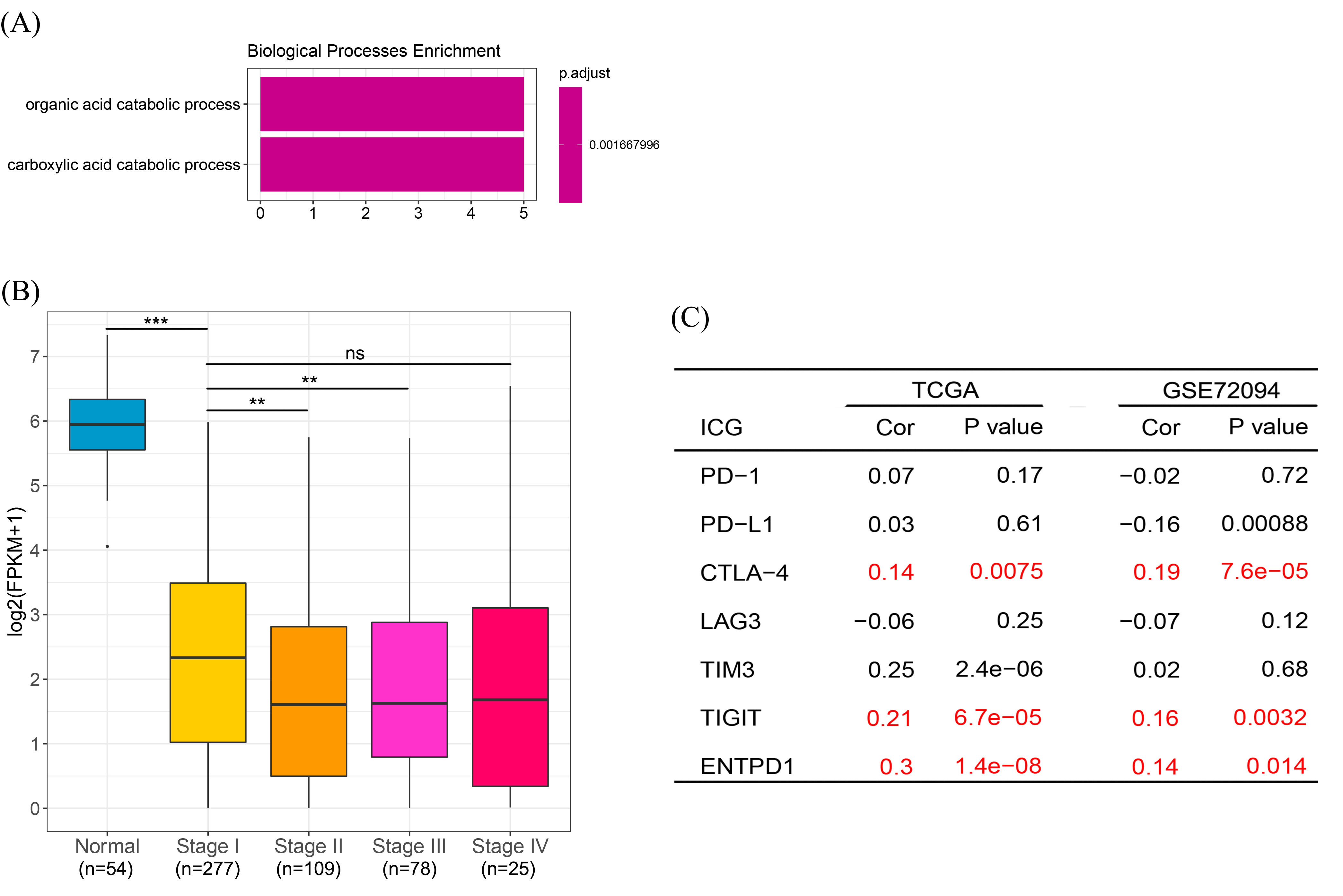


**Figure S5.** (A) Enrichment analysis of biological process of the metabolism-related genes in the MRGPI model. (B) Boxplots of the expression level of ADH1B in the normal tissue and different tumor stages. (C) Pearson’s correlation test between ADH1B and immune checkpoint genes.


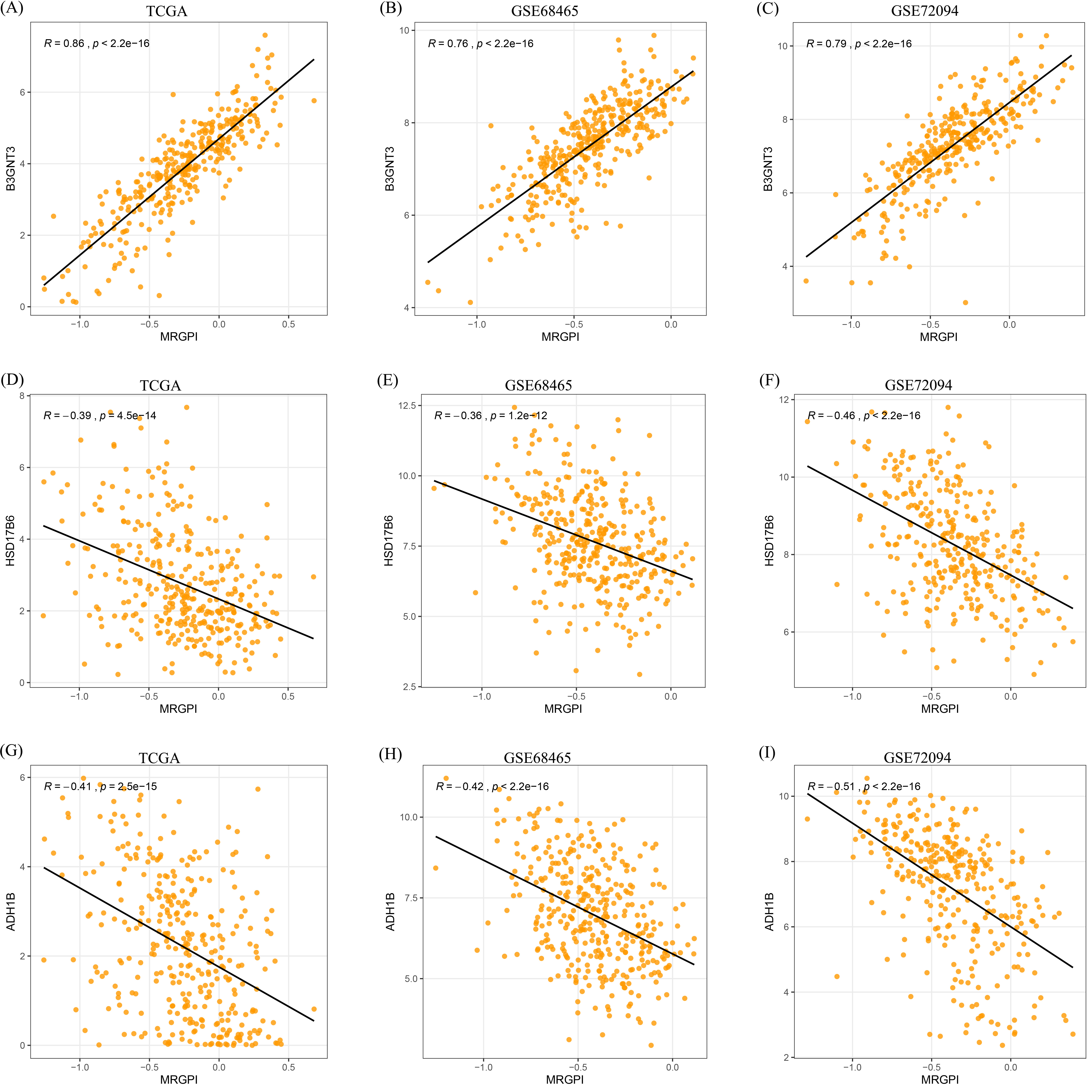


**Figure S6.** Pearson’s correlation test between the expression level of B3GNT3 (A-C), HSD17B6 (D-F), ADH1B (G-I) and MRGPI in the TCGA, GSE68465 and GSE72094 datasets, respectively.


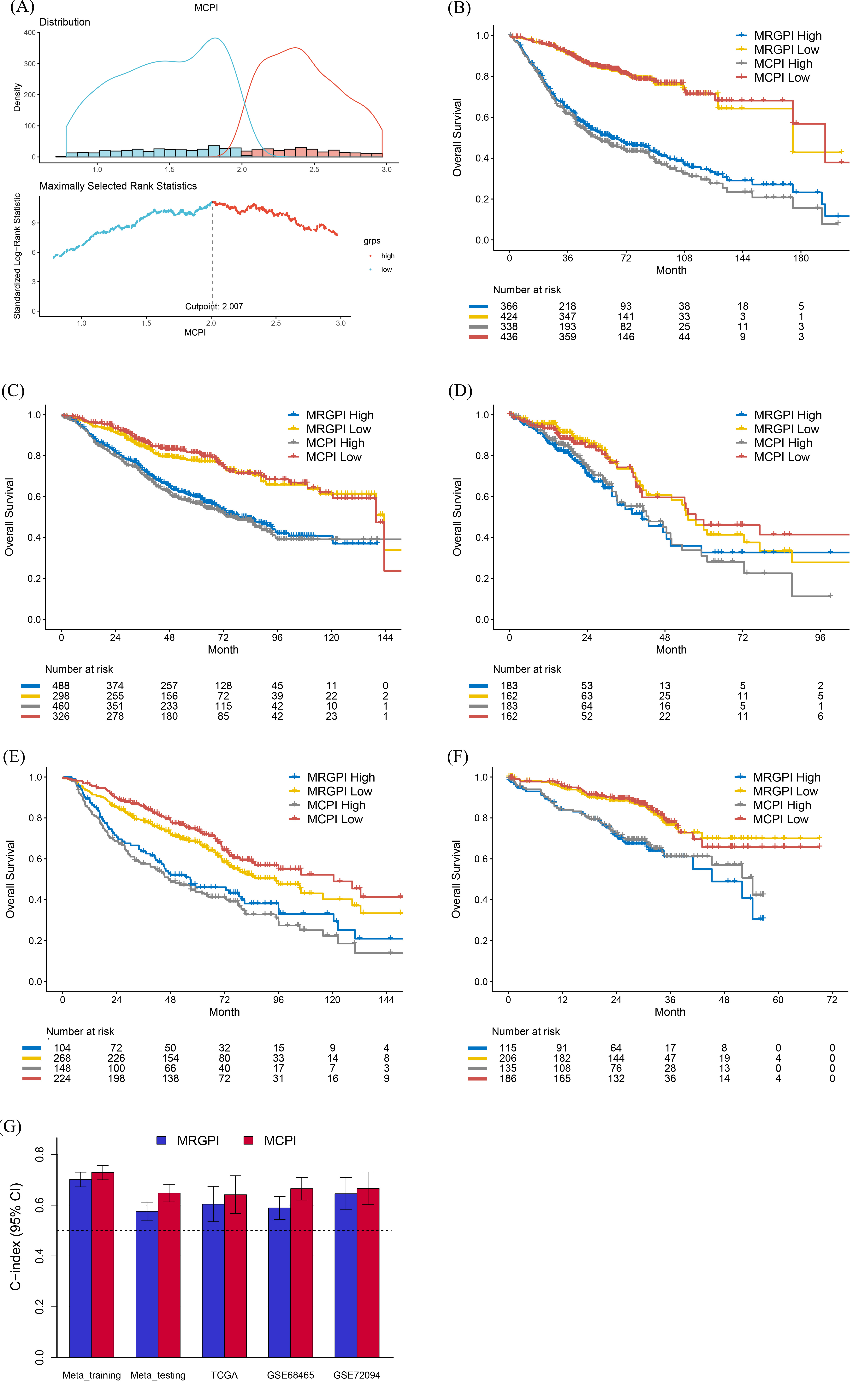


**Figure S7**. (A) Optimal cutoff value of MCPI selected by the maximally selected rank statistics. (B-F) Kaplan-Meier curves for overall survival of all patients stratified by the MRGPI and the MCPI in the meta-training (B), meta-testing (C), TCGA (D), GSE68465 (E) and GSE72094 (F) cohorts. (G) C-index comparison between MRGPI and MCPI in all cohorts.
